# The Over-Feeding of Infants

**Published:** 1901-09-14

**Authors:** Eric Pritchard


### HOSPITAL CLINICS AND MEDICAL PROGRESS.

### THE OVER-FEEDING OF INFANTS.

Abstract of a paper read before the British Medical Association Annual Meeting, Cheltenham, 1901, by ERIC PRITCHARD, M.A., M.D.Oxon., M. R.C.P.Lond.

I PROPOSE briefly in this paper to indicate the manner in which the strain of over-feeding may fall upon the various organs of an infant, and the pathological consequences of its incidence in individuals with unequal powers of resistance, and different reserve forces. For the sake of convenience I propose to classify the resulting disturbances into three groups, *i.e.* :—

1. The disturbances due to the mechanical effects.
2. The disturbances due to interference with the primary or gastro-intestinal digestion.
3. The disturbance due to interference with secondary digestion, or the general metabolic processes of the tissues.

As regards the disturbances belonging to the first group, I will dismiss them as shortly as possible, not because they are unimportant, but because they are more generally recognised. First and foremost must be mentioned dilatation of the stomach from over-filling and incomplete emptying, with consequent fermentation of the contents, bringing in its train irritation and catarrh of the mucous membrane, spasm and hypertrophy of the pyloric sphincter, retention of food and vomiting, and auto-intoxication from absorption of the product of fermentation. These evil consequences act and react on one another in an ever-widening circle, and lead finally to marasmus. Next I would briefly indicate the effect upon the intestines. The development of the intestine within limits depends on its functional activity. When the stomach permits of the passage of large quantities of food, and an undue amount enters the intestines, they develop in a corresponding ratio, and according to Marfan may attain proportions which may be nearly 100 per cent. in excess of their normal length. This is particularly the case in rachitic infants with a pot belly, and in those who have suffered habitually from gastro-intestinal troubles. Undue length of the intestine probably constitutes no unimportant factor in the determination of intussusception, volvulus, hernia and enteroptosis. It indubitably leads to intestinal inertia, and to the production of diarrhœa alternating with constipation. The disadvantages of a long intestine have recently been clearly emphasised by Metchnikoff, because such a condition admits of a large and varied bacterial flora with its attendant evils. Further than this a long intestine interferes with the descent of the diaphragm, and consequently the oxygen supply, and mechanically with the normal development of the bony parietes of the chest.

Now as regards the disturbances of gastro-intestinal or primary digestion. It may be sufficient to say that if the food be so excessive as to dilute the natural juices to a point at which they are no longer physiologically effective, it—that is to say, the food—becomes the legitimate pabulum of various forms of micro-organisms. The embarrassments of the digestive functions are the opportunities of parasitic bacteria. The products of their activity lead to every variety of auto-intoxication, especially to those which show a selective preference for the nervous system, and among these one includes eclampsia infantum, tetany, and epilepsy. The various auto-intoxications from fermentative and putrefactive processes in the gastro-intestinal tract are now sufficiently well recognised to need no further comment, but I would once again emphasise the suggestion that excess of food beyond the physiological requirements of the organism, is the chief cause of these toxæmias; directly by supplying a surplus nutritive media for the growth of parasites, and indirectly by leading to increased length of the intestine.

To pass now to the important effects of overfeeding upon the tissues.

It is a biological law, which admits of no exception, that over-stimulation leads to fatigue and loss of function. It must follow, therefore, that the liver, for instance, if over-stimulated in its special function, must show signs of fatigue. Clinically this is seen in the case of this organ, when the stools become pale and greasy, or when surplus sugar begins to appear in the urine. Both of these conditions can be cured with the greatest ease in the initial stages by giving the liver rest—in other words, by limiting or withholding food. If allowed to continue untreated, the disability of the organ may become permanent. Alimentary glycosuria, so-called, may run on to a serious form of pernicious diabetes. I see that three cases of this kind in children have recently been reported in America, and attributed to the abuse of a proprietary food, and I myself know of a similar case in this country in which there was gross abuse of sugar followed by glycosuria and finally diabetes. There is apparently a very great distinction in the liability of different children to show evidence of alimentary glycosuria, they possess what is called a different *limit of utilisation* or *co-efficient of assimilation*; rachitic children, for instance, will pass sugar in the urine after the ingestion of quantities of this carbohydrate, which will have no effect at all on healthy individuals. This has been clearly shown by Nobecourt. I have never yet seen a rachitic child whose history, when a history was to be attained, did not show that there had been over-feeding with carbohydrates. In most cases the over-feeding is absolute, in some merely relative. But this pronounced glycolytic disability on the part of rachitic children is surely a clear indication that carbohydrates should only be employed in very small quantities.

If, then, the troubles of over-feeding, whether relative or absolute, are to be avoided, it is necessary to discover what really are the true physiological requirements for each particular case.

I think it may be accepted that the standard of quantities drawn up by Drs. Rotch and Holt, form a very reliable basis for the feeding of strong, healthy infants. But in proportion as the vital index and the limit of utilisation of an infant fall short of the normal standard, so should the food supply fall short of what is indicated in the case of a strong and healthy infant.

No exact rules can be formulated for the purpose of arriving at a physiological dietary. Experience is the most important guide; examination of the increment in weight, of the stools and of the urine, are of course useful indications; but the family and hereditary history is quite as important. Children of syphilitic parents, of parents suffering from gout, rheumatism, phthisis, alcoholism, mental disease, and many other morbid conditions, are brought into this world with powers of metabolism and "*co-efficients of assimilation*" which are always low, and sometimes almost reduced to zero. Such children can be steered through the dangers of childhood on diets, which, if examined in the police courts, might render the parents liable to conviction for deliberate starvation. I have kept infants answering to this description alive and in health for months on dietaries less than one-third that of the standard of requirements according to Rotch for healthy infants, and these children have shown a slow but progressive gain of weight.

